# Effects of Honey on Oral Mucositis among Pediatric Cancer Patients Undergoing Chemo/Radiotherapy Treatment at King Abdulaziz University Hospital in Jeddah, Kingdom of Saudi Arabia

**DOI:** 10.1155/2017/5861024

**Published:** 2017-02-07

**Authors:** Soad K. Al Jaouni, Mohammad S. Al Muhayawi, Abear Hussein, Iman Elfiki, Rajaa Al-Raddadi, Saad M. Al Muhayawi, Saad Almasaudi, Mohammad Amjad Kamal, Steve Harakeh

**Affiliations:** ^1^Department of Pediatric Hematology/Oncology, King Abdulaziz University Hospital (KAUH), Faculty of Medicine, King Abdulaziz University (KAU), Jeddah, Saudi Arabia; ^2^Yousef Abdullatif Jameel Chair of Prophetic Medicine Application (YAJCPMA), King Abdulaziz University Hospital (KAUH), Faculty of Medicine, King Abdulaziz University (KAU), Jeddah, Saudi Arabia; ^3^Saudi Ministry of Health, Jeddah, Saudi Arabia; ^4^Department of Head & Neck Surgical Oncology, KAUH, Faculty of Medicine, KAU, Jeddah, Saudi Arabia; ^5^Biology Department, KAU, Jeddah, Saudi Arabia; ^6^King Fahd Medical Research Center (KFMRC), KAU, Jeddah, Saudi Arabia; ^7^Enzymoics, 7 Peterlee Place, Hebersham, NSW 2770, Australia; ^8^Novel Global Community Educational Foundation, Hebersham, NSW 2770, Australia; ^9^Special Infectious Agents Unit, KFMRC, KAU, Jeddah, Saudi Arabia

## Abstract

One of the most common complications of cancer chemotherapy is oral mucositis. This study evaluates the therapeutic effects of honey with the focus on grade III and IV oral mucositis, reduction of bacterial and fungal infections, duration of episodes of oral mucositis, and body weight in pediatric leukemic patients undergoing chemo/radiotherapy. This is an open labeled randomized controlled study conducted at our hospital on 40 pediatric cancer patients undergoing chemo/radiotherapy. All the 40 patients included in this study experienced a sum total of 390 episodes of fever and neutropenia associated with oral mucositis. A significant reduction of oral mucositis, associated* Candida*, and aerobic pathogenic bacterial infections was noted in patients in the honey treatment group. Also, there is a significant decrease in the duration of hospitalization for all those in the treatment group combined with a significant increase of body weight, delayed onset, and decreased severity of pain related to oral mucositis. Complications of oral mucositis can be tremendously reduced by the topical application of local Saudi honey and honey should be used as an integrative approach in prophylaxis and treatment of chemo/radiotherapy-induced oral mucositis in pediatric cancer patients. Further research is needed to elucidate and better understand the underlying mechanism.

## 1. Introduction

Mucositis is considered as one of the most common oral problems associated with cancer therapy [1]. Mucositis causes inflammation and ulceration of the oral cavity mucosa and be more susceptible to infection which may result in the demise of the patient due to infections and compromising the cancer treatment. Around 40%–76% of cancer patients undergoing high dose of chemotherapy and radiotherapy develop mucositis which manifests itself as intense erythema in the treated areas and patients suffer from difficulties with swallowing [1–3]. In general, the incidence rate of mucositis is two to three times higher in patients with blood malignancies associated with bone marrow suppression like lymphoma [4]. Younger cancer patients undergoing chemotherapy are more at risk of developing mucositis and may reach 90% in children under 12 years of age [5]. Some degree of mucositis manifests itself in almost all (nearly 90% to 97%) cancer patients undergoing radiotherapy [6, 7]. Among those patients around 34% to 43% showed severe mucositis [5]. As a consequence to that, patients will suffer from infections caused by both Gram positive and negative bacteria as well as fungi like* Candida* [7]. In addition, the patient's quality of life will be affected, hospital admittance rates will be higher, the use of total parenteral nutrition will be increased, and interruption of treatment will be more frequent, all of which compromise the treatment of cancer [5]. Cancer treatment will be much more effective if it is not associated with short and long term side effects as those associated with oral mucositis. Oral mucositis has also a major impact on the quality of life and nutritional status, prolonged hospital stays, and severe infections. Management essentially consists of pain management, with topical and oral analgesics/anesthetics and anti-inflammatory agents, and systemic use of antifungal medications [8]. In spite of the fact that there are many positive trials, none of those showed overwhelming data to strongly support the use of a certain agent for the treatment of oral mucositis [9].

Currently, the only standard oral hygiene consists of an oral rinse of warm water, salt, and baking soda 4 times a day. Basic oral care (brushing and flossing as tolerated) is recommended to maintain general mucosal health and to reduce the impact of oral microbial flora [10]. Some recent published data showed that honey has a positive effect against oral mucositis [11]. This study was undertaken to evaluate the efficacy of using local Saudi honey as integrative approach in prophylaxis and treatment of chemo/radiotherapy-induced oral mucositis (grades III and IV) among pediatric cancer patients in the cancer ward at King Abdulaziz University Hospital (KAUH). Other parameters were also monitored which are indicative of the success of the integration of honey with ongoing treatment of those patients and they include bacterial and fungal infections, duration of episodes of oral mucositis (as evaluated by the length of hospital stay per episode), and body weight.

## 2. Materials and Methods

### 2.1. Design and Setting

This was an open labeled randomized controlled study carried out on 40 patients in the pediatric cancer ward at KAUH, Jeddah, KSA, for a period of one year comparing the efficacy of the consumption of local Saudi commercial honey on chemo/radiotherapy-induced oral mucositis among various pediatric cancer patients who have hematological (acute lymphoblastic leukemia (ALL), acute myeloid leukemia (AML), Burkett's lymphoma, and langerhans cell histiocytosis) and nonhematological (Wilms' tumour, neuroblastoma, and medulloblastoma) cancer.

### 2.2. Participants

**Figure 1:**
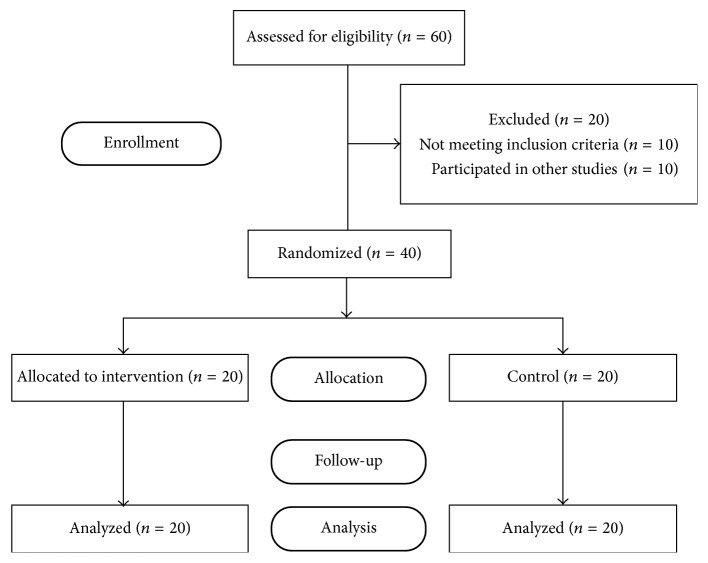
Patient enrollment.

Sixty patients were admitted to oncology ward diagnosed for both hematological and nonhematological cancer, forty of whom fulfilled the inclusion criteria and as such were included in this clinical trial. The duration of the study was one year (Figure 1).

Patients were assessed for oral mucositis prior to chemotherapy courses and daily during episodes of admission for supportive care of febrile neutropenia. All patients were encouraged to apply hospital provided honey to all areas of oral mucosa, gingiva, and tongue followed by mouth rinsing with alkaline saline, four to six times daily. Clinical assessment was done by attending physicians, nurses, and dentists whenever needed. Normal oral mucosa was defined by pink, moist appearance with no lesions, crusts, or debris. Normal gingiva was recognized by being pink and firm. Patients with healthy oral cavity were still counseled and encouraged to keep up their oral hygiene regimen including local mouth application of hospital honey. The following grading system was used to assess severity of oral mucositis [2]. Grade I oral mucositis was defined with shiny red oral mucosa and/or gingiva with possible swelling and white patches with possibly red coated swollen tongue; patients with grade I oral mucositis may complain of a burning sensation or gingival discomfort. Grade II oral mucositis was defined by same mucosal and gingival findings previously described in grade 1 with added painful ulcers; patients can still tolerate solids and liquids. Grade III oral mucositis was defined with severe erythema, ulceration, or white patches over oral mucosa with severe pain; patient cannot tolerate solid diet but can tolerate fluids only. Grade IV oral mucositis was defined with severe erythema, ulcerations, and white plaques that affect oral intake for both solid and fluid diets even drooling of saliva. Various indicators which are involved in bacterial and fungal infections were monitored. For bacterial infections, aerobic cultures were checked and* Candida* colonization was monitored as an indicator of fungal infections. Inclusion criteria: Pediatric cancer patients at KAUH above 1 year of age treated with chemo/radiotherapy whose parents or their assigned care takers approved them to participate in this study signed a consent form. Exclusion criteria: They include pediatric cancer patients at KAUH who were less than one year old and also those patients who refused to participate in this study or had allergy to honey. Those who were eligible and agreed to participate were invited to our clinic with their parents to get information about them. Such information included the following: sex, age, body weight, educational level, occupation, and their records checked for the presence of any other systemic diseases. They all underwent a physical examination of the mouth and throat for any abnormalities. Routine laboratory tests were conducted. Those patients with other systemic disease were not included in this study.

### 2.3. Aerobic Bacterial Test and* Candida* Assay

This was done by taking oral and oropharyngeal swabs. Swabs were sent to the KAUH clinical laboratory for assay. Oral swabs were collected by gently rubbing a sterile cotton swab over the labial mucosa, tongue and cancerous lesion [12]. After the swabs were collected, they were inoculated onto sheep blood agar, Sabouraud dextrose agar, MacConkey agar, nutrient agar, and other selective media and then incubated under aerobic conditions for 24–48 hours at 37°C temperature for bacterial pathogens isolation and for 24–72 hours at 30°C in BOD incubator for fungal species isolation [13].

### 2.4. Intervention

All of the 40 patients were randomly distributed into two groups, each containing 20 patients of both sexes. Patients in both groups then received chemo/radiotherapy in addition to the routine oral hygiene (Lidocaine, Mycostatin, Daktarin mouth gel, and mouth wash).

The experimental group received topical application of pure natural honey as prophylaxis before the development of oral mucositis or during the episodes of fever and neutropenia associated with oral mucositis. Local commercial Saudi honey bought from the supermarkets was used.

### 2.5. Evaluation of Outcomes

The severity of oral mucositis was described according to the World Health Organization's oral toxicity scale. Grade I: soreness ± erythema, grade II: erythema, ulcers, and patients' ability to swallow solid foods, grade III: ulcers with extensive erythema and patients not being able to swallow solid foods, and grade IV: mucositis to the extent that alimentation is not possible [2]. Oral mucositis was evaluated before and after treatment and also a week after commencing treatment [14]. The duration of stay in the hospital per episode was reported, in addition, to monitor the fluctuations of the body weight in those patients.

### 2.6. Statistical Analyses

All data was entered using SPSS 17 software (SPSS Inc., Chicago, IL) and analyzed. The data were double checked and cleaned and analyzed in terms of frequencies. Continuous variables were presented as mean and standard deviation (STD) and categorical variables were presented as absolute and relative frequencies. Independent test and Chi-square test were used to investigate whether there was significant difference between the treatment and experimental groups.

Absolute Risk Reduction (ARR) and Number Needed to Treat (NNT) together with the 95% Confidence Interval (95% CI) were presented. *P* values <0.05 were considered to be significant.

### 2.7. Ethical Approval

The patients and those who were in charge of them were informed about the objective of this study and the resulting possible benefits, the prescribed ways, and their own role. An informed consent form was signed just before enrolling patients in the study. All personal data was kept confidential. This study design was approved by the Ethical Committee at King Abdulaziz University.

## 3. Results

The 40 patients experienced episodes of fever and neutropenia associated with oral mucositis. Most of those included were hematological patients (90% in the honey treated group versus 75% in the control group). The characteristics of the patients involved are shown in Table 1.

Both sexes were included in the treatment and control groups with an average age of about 8 years (SD ± 4.2) in both groups (Table 1). There was no significant difference between the control and the honey treated group in relation to gender or age.  Table 2 shows the Absolute Risk Reduction and Number Needed to Treat for developing grade III and IV oral mucositis,* Candida*, and aerobic bacterial infections. The results show a significant difference between the experimental (honey) and the control group (*P* < 0.05).

The results showed a significant ARR in grade III and IV oral mucositis of 35% in the treatment group (*P* = 0.02) with an NNT of 2. The same was also true in the case of* Candida* colonization with an ARR of 50% in the treatment group (*P* = 0.003) with an NNT of 2. In the case of the aerobic plate count, there was also a significant ARR of bacteria of 50% in the treated group versus the control with an NNT of 2 (*P* = 0.003) (Table 2).

The study, also, showed significant reduction in the duration of hospitalization for oral mucositis patients in the treatment group as compared to the control group. The mean days for hospitalization were 7 ± 3 days/episodes and 13 ± 5 days/episodes for the treatment group and the control group, respectively.

Patients in the treatment group had significant increase in body weight, delayed onset, and decreased severity of pain related to oral mucositis in comparison to those in the control group. The treatment group showed better improvement in all of the outcome variables.

## 4. Discussion

The results of this study indicated that there was a statistically significant reduction in the number of episodes of oral mucositis, bacterial and fungal infections, and hospital stay among pediatric cancer patients undergoing chemo/radiotherapy who are taking honey in conjunction with their regular therapy. Such an overall improvement was also accompanied by body weight gain in patients in the experimental group in comparison to the control group (Table 3).

Honey is an age-old remedy from the time of Egyptian civilization; mentioned in the holy Qur'an and more recently it has found a place in modern medicine [3, 15, 16].

The results of this study showed that honey had very positive results against oral mucositis among pediatric cancer patients undergoing chemo/radiotherapy. The results showed a significant reduction in grade III and IV oral mucositis in the experimental group (20%) incidence rate versus 55% in the control. Thus yielding a 35% ARR in the treatment group (*P* = 0.02) with an NNT of 2. Those results are in agreement with what was reported by others on the use of honey inside the mouth of cancer patients undergoing chemo/radiotherapy. A higher reduction of 80% in radiation-induced oral mucositis was noted when honey was applied inside the mouth of patient's treatment, directly after and few hours after treatment [17]. Similar results of 20% of participants in experimental group developed grade III or grade IV mucositis, in a study which evaluated the effects of application of honey in management of radiation-induced mucositis, as compared to 75% of participants in control group [18, 19]. In another study regarding the application of honey to prevent radio chemotherapy induced oral mucositis. It was reported that none of the patients in the experimental group developed grade IV mucositis. However, only three patients in the experimental group developed grade III mucositis. This is in contrast to 13 patients in control group who developed grade III or grade IV mucositis [20]. In a different study, one subject in the experimental group developed grade III oral mucositis in comparison to 8 subjects in control group who developed grade III oral mucositis. None in the experimental group developed grade IV oral mucositis [21].

In a single blinded experimental study aimed at evaluating the effect of honey on irradiation induced mucositis, it was noted that there was significant reduction in the degree of oral mucositis in experimental group as compared with control group [22]. In current study, there was a significant reduction in the number of episodes of oral mucositis between the experimental versus the control group. 20% of the patients in the experimental group had developed grade III and IV oral mucositis during the one year of the study in comparison to 55% in the control group.

Honey seemed to enhance the efficacy of therapy in the treatment of oral mucositis as compared to the use of either honey alone or steroids [11, 23–25]. In a randomized controlled study on the effects of honey on oral mucositis, it was noted that there was a statistically significant difference between the experimental and the control group in weeks 4, 5, and 6. For instance, only 7.14% of the participants in the experimental group developed mucositis in comparison to 64.28% in the control group who did not take honey [21].

Honey has long been known to have a soothing action on mucus membranes and recommended for the management of oral mucositis. Honey is the by-product of flower nectar. Because of its high viscosity, acidic PH, hydrogen peroxide, high osmolarity, and rich nutritional properties, honey can inhibit bacterial and fungal growth [7, 26, 27] and enhance healing and is thereby a justified approach in the management of oral mucositis [16].

Infection damaged mucosal tissues are more susceptible to developing a wide variety of bacterial (due to loss of normal tissue response), viral (herpes), and fungal infections. In addition to the impaired effect of the normal immune response caused by decreased saliva volume, alterations in saliva quality and decreased levels of immunity. Such changes result in an increase in the dominance of opportunistic pathogenic organisms at the cost of the normal oral microbiota which are the good bacteria that offer protection [28]. Septicemia may develop among those patients and may be life threatening infection [29]. Numerous studies have reported that Gram negative bacterial flora of the oropharynx dominates in patients during myelosuppression and in those who are receiving head and neck irradiation [30]. As a result, those colonizing Gram negative bacteria oral microflora may release endotoxins, known to be potent inflammation inducers, thus leading to a cascade of inflammation processes and further intensify the patients' local mucosal injury [15]. Accordingly, it has been hypothesized that oral mucositis may be reduced by using specific antimicrobial therapy against those dominating bacteria [3].

Fungal infections are also common among those patients; particularly Candidiasis, caused by* Candida albicans*, is a common fungal infection present among such patients and is known as oral thrush. Such a symptom is painful and associated with erythema or discrete white plaques and may be easily confused with mucositis. The taste buds in the mouth are affected by radiation and as such they may become impaired as a result and cause changes in taste sensations [28, 31]. Such taste changes may be related to saliva which may modulate some of the tastes (sour, bitter, salt, and sweet) through biochemical interactions [31]. Alteration in the taste will affect the appetite which will be compromised as a result, thus affecting the nutritional status and quality of life of the patient. Nausea and vomiting are a common occurrence among those patients receiving chemo/radiotherapy. Nausea, vomiting, and taste changes affect the ability to eat, tolerate certain foods, and eat less and do not harvest full energy from the entire food consumed and as such lead to a reduction in the patient's body weight.

Our data showed a significant weight gain in the honey treated group versus the control group. The results of a comparative study on the evaluation of honey versus sucralfate against oral mucositis indicated the mean weight loss was more in sucralfate group as compared to honey and it was concluded that honey was more effective in increasing the weight as compared to sucralfate group [32]. Also, the results of another randomized single blind study showed that there was more weight loss in those in the control as compared to the honey treated group [22, 32, 33]. It was reported that patients treated with topical honey showed that 71% of the treated group showed no weight loss as compared to 22% in the control group [19, 32, 34–37]. Oral mucositis is normally associated with pain which results from the loss of the epithelial lining, ulceration and the associated edema. Also pain results from the neurotransmitters related to the inflammatory response associated with oral mucositis [28]. The pain becomes more intense when the pharyngeal mucosa is affected and results in burning sensations experienced by the patient upon swallowing.

Pediatric cancer patients have poor nutritional status before starting chemo/radiotherapy treatment and it decreases with a number of mucositis related side effects such as dysphagia and the loss of taste and saliva. Also, feeling down affects the appetite [38]. Inadequate nutrition leads to weight loss and such patients may require other means of nutrition. Salivary secretions are also reduced [39–41]. Such a decrease will result in dryness of the mouth and causes oral discomfort, altered taste, nutritional impairment (difficulty in mastication and swallowing), and dental decay.

The data obtained in this study revealed that there was a significant reduction in the days of hospitalization during an oral mucositis episode in the experimental group in comparison to the control group throughout the one-year duration of the study. In a study which took place for 4 weeks and indicated that 21.42% of patients in control group were hospitalized due to severe mucositis. This is in contrast to none in the experimental group were hospitalized due to severe mucositis. Due to the development of severe oral mucositis in five patients in the control group were treated, while none in experimental group had treatment interruptions. The results of another study revealed that 16% of patients who received radiotherapy were hospitalized due to severe mucositis. In addition to having unplanned break in the treatment protocol was also reported in 11% of patients in the same study [42].

In spite of the fact that the underlying mechanism of action of honey is not well elucidated, it is likely that factors like osmolality, phenol content, flavanoid levels, acidity, and the release of hydrogen peroxide are thought to be the most important factors for its activity [43]. Honey is known for its antioxidant and anti-inflammatory activities and the increase of nitric oxide (NO) in the lesions [25, 43, 44]. Being sweet, honey may per se stimulate the salivation reflex due to their hyperosmolarity. As such its efficacy may be related to its hyperosmolarity, anti-inflammatory, and antioxidant properties [34, 45–48]. As a consequence, to all those, honey may accelerate the repair and healing of mucosal damage and reduce associated irritations [11, 49, 50].

*Limitations*. A larger sample size is recommended for future studies for further validation of the results.

## 5. Conclusions/Recommendations

This study showed that the topical honey treatment is effective in reducing and minimizing oral mucositis among pediatric cancer patients treated with chemo/radiotherapy and is cost-effective treatment. It also showed a reduction in hospitalization duration, reducing painful mucositis, and increasing body weight. Honey is a natural product, is cheap, has less side effects, is tolerated well by most of the patients, and has a delicious taste. We recommend using topical honey as a part of the standard supportive care for chemo/radiotherapy-induced oral mucositis in pediatric cancer patients. The results warrant further investigation.

## Figures and Tables

**Table 1:** Characteristics of study participants.

	Treatment (honey)	Control	*P* value
Gender			
Boys	11 (55)	10 (50)	0.7
Girls	9 (45)	10 (50)	
Age	7.9 (4.1)	8.1 (4.9)	0.8
Diagnosis			
Hematological	18	15	0.4
Nonhematological	2	5	

**Table 2:** Absolute Risk Reduction (ARR) and Number Needed to Treat (NNT) for developing grade III and IV oral mucositis, *Candida*, and aerobic bacterial infections with 95% CI between honey and control group.

	Honey (*n* = 20)	Control (*n* = 20)	ARR^*∗*^ (95% CI)	NNT^*∗∗*^ (95% CI)	*P* value
Grade III and IV mucositis	4 (20)	11 (55)	35 (9.6–61.7)	2 (2–10)	0.02
*Candida*	2 (10)	12 (60)	50 (20.7–69.5)	2 (1–5)	0.003
Aerobic plate count	2 (10)	12 (60)	50 (20.7–69.5)	2 (1–5)	0.003

^*∗*^Absolute Risk Reduction; ^*∗∗*^Number Needed to Treat.

**Table 3:** Effect of honey on the duration of hospitalization per episode of oral mucositis and on the % body weight gain.

	Honey (*n* = 20) mean (SD)	Control (*n* = 20) mean (SD)	Mean difference	*P* value^*∗*^
Hospitalization (days/episode)	7 (3)	13 (5)	−4.6	<0.001
Percentage increase in body weight (%)	35.1 (6.5)	15 (4.2)	19.9	<0.001

^*∗*^ *t*-test.
